# Longitudinal Tau PET Using ^18^F-Flortaucipir: The Effect of Relative Cerebral Blood Flow on Quantitative and Semiquantitative Parameters

**DOI:** 10.2967/jnumed.122.263926

**Published:** 2023-02

**Authors:** Denise Visser, Hayel Tuncel, Rik Ossenkoppele, Maqsood Yaqub, Emma E. Wolters, Tessa Timmers, Emma Weltings, Emma M. Coomans, Marijke E. den Hollander, Wiesje M. van der Flier, Bart N.M. van Berckel, Sandeep S.V. Golla

**Affiliations:** 1 Department of Radiology and Nuclear Medicine, Amsterdam Neuroscience, Vrije Universiteit Amsterdam, Amsterdam UMC, Amsterdam, The Netherlands;; 2 Alzheimer Center Amsterdam, Department of Neurology, Amsterdam Neuroscience, Vrije Universiteit Amsterdam, Amsterdam UMC, Amsterdam, The Netherlands;; 3 Clinical Memory Research Unit, Lund University, Lund, Sweden; and; 4 Department of Epidemiology and Biostatistics, Vrije Universiteit Amsterdam, Amsterdam UMC, Amsterdam, The Netherlands

**Keywords:** Alzheimer disease, dynamic (DVR/BPND), longitudinal 18F-flortaucipir PET, quantification, static (SUVr)

## Abstract

Semiquantitative PET measures such as SUV ratio (SUVr) have several advantages over quantitative measures, such as practical applicability and relative computational simplicity. However, SUVr may potentially be affected by changes in blood flow, whereas quantitative measures such as nondisplaceable binding potential (BP_ND_) are not. For ^18^F-flortaucipir PET, the sensitivity of SUVr for changes in blood flow is currently unknown. Therefore, we compared semiquantitative (SUVr) and quantitative (BP_ND_) parameters of longitudinal ^18^F-flortaucipir PET scans and assessed their vulnerability to changes in blood flow. **Methods:** Subjects with subjective cognitive decline (*n* = 38) and Alzheimer disease patients (*n* = 24) underwent baseline and 2-y follow-up dynamic ^18^F-flortaucipir PET scans. BP_ND_ and relative tracer delivery were estimated using receptor parametric mapping, and SUVr at 80–100 min was calculated. Regional SUVrs were compared with corresponding distribution volume ratio (BP_ND_ + 1) using paired *t* tests. Additionally, simulations were performed to model effects of larger flow changes in different binding categories. **Results:** Results in subjective cognitive decline and Alzheimer disease showed only minor differences between SUVr and BP_ND_ changes over time. Relative tracer delivery changes were small in all groups. Simulations illustrated a variable bias for SUVr depending on the amount of binding. **Conclusion:** SUVr provided an accurate estimate of changes in specific binding for ^18^F-flortaucipir over a 2-y follow-up during which changes in flow were small. Notwithstanding, simulations showed that large(r) flow changes may affect ^18^F-flortaucipir SUVr. Given that it is currently unknown to what order of magnitude pharmacotherapeutic interventions may induce changes in cerebral blood flow, caution may be warranted when changes in flow are potentially large(r), as in clinical trials.

## Visual Abstract

**Figure:**
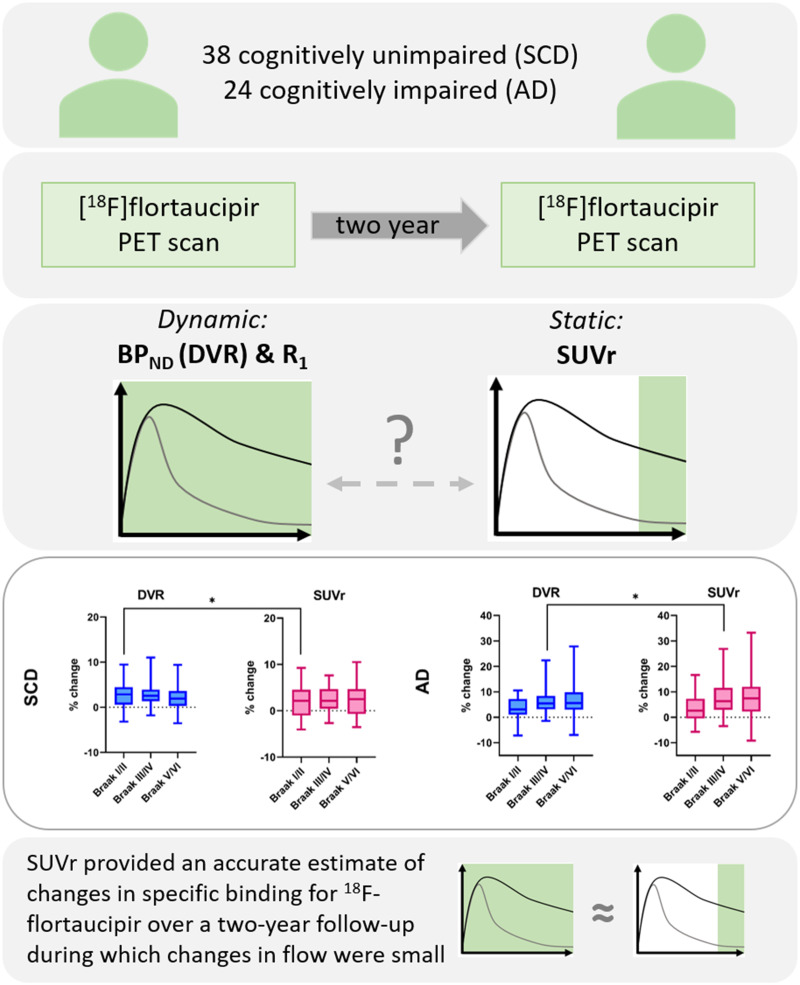

In vivo tau imaging allows for quantification of longitudinal changes in tau accumulation during the course of Alzheimer disease (AD) and can serve as a surrogate outcome measure in clinical trials. Several tau PET tracers are available for this purpose, of which ^18^F-flortaucipir is the only one approved by the Food and Drug Administration (1–5). ^18^F-flortaucipir PET images can be acquired using static or dynamic scanning protocols. Semiquantitative parameters such as SUV ratio (SUVr) can be derived from such a static PET scan. However, parameters derived from a dynamic PET scan, such as distribution volume ratio (DVR) or nondisplaceable binding potential (BP_ND_), are fully quantitative and overall more accurate (6*,*^7^). Notwithstanding, dynamic protocols—because of the long scan duration—result in patient movement, lower patient comfort, and lower scanning efficiency. A compromise can be achieved by implementing a dual-time-window protocol in which overall scanning time is reduced by introducing a resting period during the scan while maintaining high quantitative accuracy (8–10).

SUVr has the advantage of practical applicability and relative computational simplicity (2–5), while dynamic imaging studies provide more accurate measurements of specific binding and measure the relative tracer delivery (R_1_), a proxy for relative cerebral blood flow (“^18^F-flortaucipir R_1_” section in the supplemental materials available at http://jnm.snmjournals.org) (7*,*^11^–^15^). R_1_ is important because blood flow changes can occur over time in AD because of disease progression or drug intervention. Longitudinal changes using SUVr may be biased by blood flow changes, whereas quantitative measures (BP_ND_) are not (6*,*^16^). Currently, for ^18^F-flortaucipir the sensitivity of SUVr for changes in blood flow has not been investigated. Therefore, with this study we compared SUVr and DVR/BP_ND_ for ^18^F-flortaucipir PET in a 2-y follow-up observational study. Second, we used simulations to investigate how larger changes in R_1_ affect SUVr and DVR/BP_ND_.

## MATERIALS AND METHODS

### Participants

We included 62 subjects from the Amsterdam Dementia Cohort (17*,*^18^), of whom 38 were cognitively normal with subjective cognitive decline (SCD) and 24 cognitively impaired (i.e., mild cognitive impairment (MCI) due to AD (19) [*n* = 4] or probable AD dementia (20) [*n* = 20], grouped into 1 MCI/AD group).

Twelve of 38 SCD subjects were classified as amyloid-β (Aβ) PET–positive (^18^F-florbetapir visual assessment (21)). All MCI/AD patients were classified as Aβ-positive by cerebrospinal fluid biomarkers (i.e., cerebrospinal fluid Aβ1-42 < 813 ng/L (22)) or a Aβ PET scan (^11^C-PiB or ^18^F-florbetaben) by visual assessment (23*,*^24^).

The study protocol was approved by the Medical Ethics Review Committee of the Amsterdam UMC VU Medical center. All patients provided written informed consent before study participation.

### Imaging

All subjects underwent 2 dynamic ^18^F-flortaucipir PET scans, acquired on a Philips Ingenuity TF-64 PET/CT scanner, with a time period of 2.1 ± 0.3 y (SCD) or 2.2 ± 0.3 y (AD) between both scanning sessions. For SCD subjects, each scanning session consisted of 2 dynamic PET scans of 60 and 50 min, respectively, with a 20-min break in between (14*,*^25^). For AD patients, each scanning session consisted of 2 dynamic PET scans of 30 min and 20 min, respectively, with a 50-min break in between (9).

BP_ND_, R_1_, and SUVr at 80–100 min were extracted in a priori–defined regions of interest (ROIs) in subject space using the Hammers and Svarer templates: Braak I/II (entorhinal), Braak III/IV (limbic), and Braak V/VI (neocortical). These ROIs align with neuropathologically defined regions (26) and are informative for tau PET in AD (27–30).

For each parameter and ROI, we calculated percentage change using the following formula (DVR [BP_ND_ + 1] or SUVr associated with the follow-up and baseline scans, respectively):

Percentage change=(follow-up/baseline−1)×100%

We repeated all analyses with partial-volume–corrected data using the iterative deconvolution method, as described previously (31–33).

### Statistical Analyses

To allow for direct comparison with SUVrs, DVR was used for all analyses. Paired *t* tests were performed to assess differences between parameters and time points. Pearson correlation coefficients were computed to assess the correlation between percentage change in SUVr and DVR (all ROIs combined). Bland–Altman analyses were performed to assess bias and agreement between percentage change in SUVr and DVR (all ROIs combined). Analyses were performed in R software, version 4.0.2, and GraphPad Prism, version 9.1.0.

To explore whether the required sample size for (theoretic) future trials would differ when either quantitative or semiquantitative methods are used, sample sizes were calculated using GPower, version 3.1.9.7. For these analyses, we used a range of 0.5%–10% expected change in tracer retention over time, to inform on longitudinal study designs in the context of ^18^F-flortaucipir. Sample sizes were calculated for SUVr and DVR, for all 3 ROIs (Braak I/II, III/IV, and V/VI). The differences between 2 dependent means (matched pairs) was calculated, with an α (error probability) of 0.05 and a power (1 − β error probability) of 0.80. To adhere to the typical duration of clinical trials in AD, we calculated percentage change over an 18-mo period and used those SDs as input for the sample size calculations.

### Simulations

Details on the methods used for simulations can be found in the Methods section of the supplemental materials.

## RESULTS

Patient characteristics are shown in Table 1. In both AD and SCD, ^18^F-flortaucipir SUVr were higher than DVR for all regions and at both time points (baseline and follow-up, all *P* < 0.001). Respective DVR, SUVr, and R_1_ values are shown in Table 2 (SCD subjects and AD patients) and Supplemental Tables 2 and 3 (Aβ-negative and -positive SCD subjects, respectively). The percentage overestimation of SUVr relative to DVR, for all regions and at both time points, is presented in Supplemental Table 4. Annualized percentage change in DVR and SUVr is presented in Supplemental Tables 5 and 6. No significant correlations between DVR or SUVr and R_1_ were observed in either SCD or AD patients (Supplemental Fig. 1). Partial-volume–corrected data yielded essentially similar results; therefore, only noncorrected data will be presented further in the article.

**TABLE 1.** Demographics of Study Population

Demographic	SCD (*n* = 38)	AD (*n* = 24)
Sex (*n*)		
Female	16	11
Male	22	13
Age at baseline (y)	65 ± 7	66 ± 7
Age at follow-up (y)	67 ± 7	68 ± 7
Time between PET scans (y)	2.1 ± 0.3*	2.2 ± 0.3*
MMSE at baseline	29 ± 1*	24 ± 3*
Aβ-positive^†^ at baseline (*n*)	12/38*	24/24*
Aβ-positive^†^ at follow-up (*n*)	16/38*	NA
APOE4 allele carriers (*n*)	12/38*	17/22 (2 unknown)*

* Significant differences (*P* < 0.05) between diagnostic groups.

^†^ SCD subjects were classified as Aβ-positive as evidenced by substantial Aβ pathology after 50- to 70-min SUVr ^18^F-florbetapir Aβ PET scan visual assessment, and mild cognitive impairment/AD patients were classified as Aβ-positive as evidenced by cerebrospinal fluid biomarkers for AD (i.e., cerebrospinal fluid Aβ1–42 < 813 ng/L) or positive Aβ PET (^18^F-PiB or ^18^F-florbetaben) findings by visual assessment.

MMSE = mini mental state examination; NA = not available.

Mean ± SD are provided, unless otherwise indicated.

**TABLE 2.** ^18^F-Flortaucipir DVR, SUVr and R_1_ Values for SCD Subjects and AD Patients

	DVR			SUVr at 80–100 min			R_1_		
	BL	FU	%change	BL	FU	%change	BL	FU	%change
SCD (*n* = 38)									
Braak I/II	1.039 (0.121)	1.066* (0.133)	2.56^†^ (2.85)	1.134^‡^ (0.159)	1.154^‡^^∥^ (0.158)	1.85 (3.27)	0.708 (0.041)	0.714 (0.049)	0.74 (3.96)
Braak III/IV	1.045 (0.075)	1.075* (0.098)	2.82 (2.54)	1.102^‡^ (0.103)	1.130*^‡^ (0.118)	2.47 (2.64)	0.836 (0.036)	0.842 (0.043)	0.79 (2.75)
Braak V/VI	1.042 (0.057)	1.067* (0.076)	2.33 (2.77)	1.076^‡^ (0.077)	1.096*^‡^ (0.093)	2.17 (3.29)	0.926 (0.043)	0.930 (0.049)	0.47 (2.67)
AD (*n* = 24)									
Braak I/II	1.277 (0.146)	1.321* (0.157)	3.48 (4.16)	1.426^‡^ (0.192)	1.470^‡^^∥^ (0.194)	3.25 (5.26)	0.713 (0.047)	0.706 (0.053)	−0.87 (5.26)
Braak III/IV	1.256 (0.147)	1.341* (0.185)	6.61^†^ (5.63)	1.367^‡^ (0.190)	1.471*^‡^ (0.229)	7.52 (6.66)	0.835 (0.045)	0.821^§^ (0.040)	−1.62 (3.71)
Braak V/VI	1.284 (0.222)	1.379* (0.260)	7.25 (6.85)	1.382^‡^ (0.281)	1.495*^‡^ (0.316)	8.21 (8.03)	0.904 (0.051)	0.883^∥^ (0.055)	−2.28 (3.67)

* *P* < 0.001, baseline vs. follow-up.

^†^ *P* < 0.05, percentage change in DVR vs. percentage change in SUVr.

^‡^ *P* < 0.001, DVR vs. SUVr.

^§^ *P* < 0.05, baseline vs. follow-up.

^∥^ *P* < 0.01, baseline vs. follow-up.

BL = baseline; FU = follow-up.

Mean ± SD are provided.

### Differences in ^18^F-Flortaucipir DVR, SUVr, and R_1_

#### SCD Subjects

**FIGURE 1.**
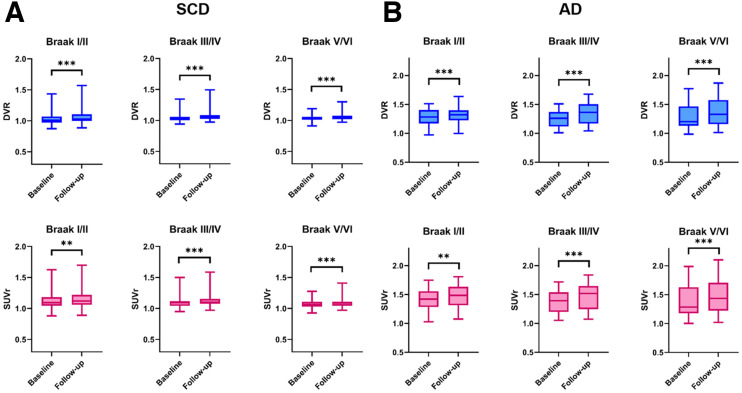
Box plots of regional DVR (upper row) and SUVr at 80–100 min (lower row) in SCD (A) and AD (B). **P < 0.01. ***P < 0.001.

**FIGURE 2.**
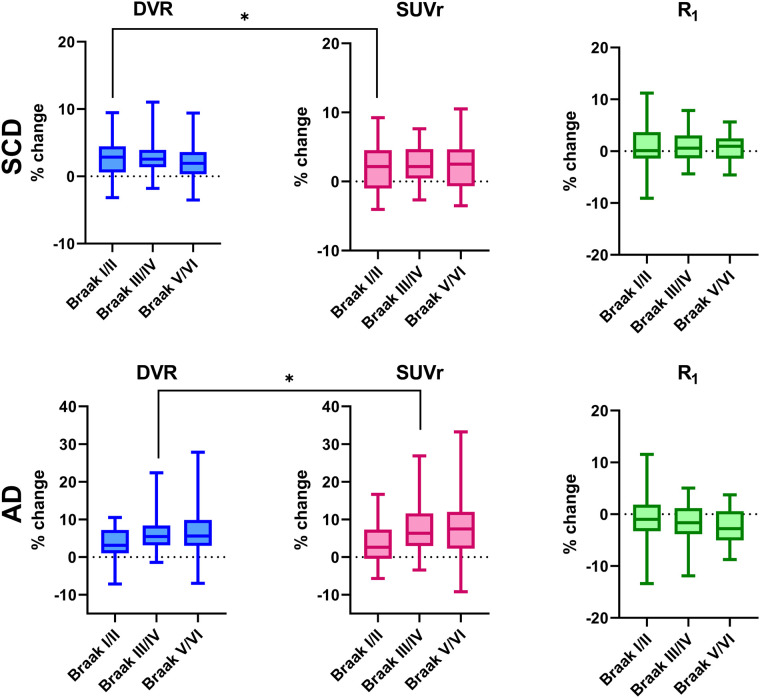
Regional percentage changes in DVR, SUVr at 80–100 min and R_1_ for SCD subjects and AD patients. *P < 0.5.

**FIGURE 3.**
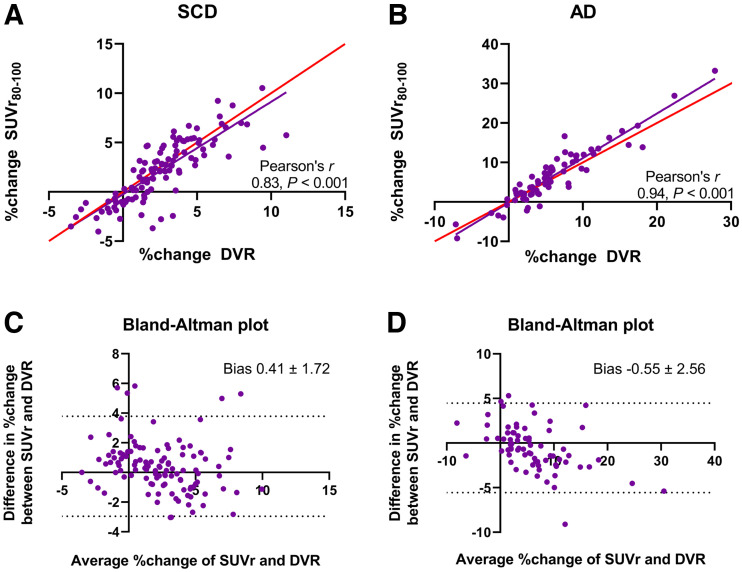
(A and B) Correlation plot of percentage change in DVR vs. SUVr at 80–100 min (SUV_80–100_) in SCD (A) and AD (B), in which red line represents line of identity. (C and D) Bland–Altman plot of percentage change in DVR vs. SUVr at 80–100 min in SCD (C) and AD (D).

DVR increased at follow-up in all regions (all *P* < 0.001), with the largest increase found in Braak III/IV (1.045–1.075, 2.82% ± 2.54%) (Table 2; Fig. 1A; Supplemental Fig. 2A). SUVr also significantly increased at follow-up in all regions (all *P* < 0.003). The largest increase was found in Braak III/IV (1.102–1.130, 2.47% ± 2.64%) (Table 2; Figs. 1C and 2C). Percentage change was significantly lower for SUVr than for DVR in Braak I/II (SUVr, 1.85% ± 3.27%, vs. DVR, 2.56% ± 2.85%; *P* = 0.048). Braak III/IV and V/VI did not show any statistically significant differences between percentage change in DVR and SUVr (Table 2; Fig. 2). Taking all regions together, the correlation coefficient between percentage change in SUVr and DVR was 0.83 (*P* < 0.001), and the bias as provided by Bland–Altman analysis was 0.41 ± 1.72 (Figs. 3A and 3C). For R_1_, no significant decreases at follow-up were found in any region (Table 2).

#### AD Patients

DVR increased at follow-up in all regions (all *P* < 0.001), with the largest increase found in Braak V/VI (1.284–1.379, 7.25% ± 6.85%) (Table 2; Fig. 1B; Supplemental Fig. 2B). SUVr also increased at follow-up in all regions (all *P* < 0.009). Like DVR, the largest increase was found in Braak V/VI (1.382–1.495, 8.21% ± 8.03%) (Supplemental Table 3; Fig. 1D; Supplemental Fig. 2D). Percentage change was higher for SUVr than for DVR in Braak III/IV (SUVr, 7.52% ± 6.66%, vs. DVR, 6.61% ± 5.63%; *P* = 0.047). No statistically significant differences between percentage change in SUVr and DVR were found for any other region (Table 2; Fig. 2). Taking all regions together, the correlation coefficient between percentage change in SUVr and DVR was 0.94 (*P* < 0.001), and the bias as provided by Bland–Altman analysis was −0.55 ± 2.56 (Figs. 3B and 3D). For R_1_, significant decreases at follow-up were found in Braak III/IV (0.835–0.821, −1.62% ± 3.71%, *P* = 0.040) and V/VI (0.904–0.883, −2.28% ± 3.67%, *P* = 0.003) (Table 2).

### Sample Size Calculations

Large differences in required sample sizes were observed for small effect sizes, with the largest differences being between methods in the AD group (Supplemental Table 7). However, with larger effect sizes (in line with expectations in clinical trials), differences in required sample size between the 2 methods became negligible for both SCD and AD (Supplemental Table 7).

### Simulations

Simulations with 5% coefficient of variance showed results similar to those for the simulated time–activity curves obtained with almost no noise (0.05% coefficient of variance). Therefore, to mimic real cohort data, only the results from time–activity curves with a 5% coefficient of variance were reported.

**FIGURE 4.**
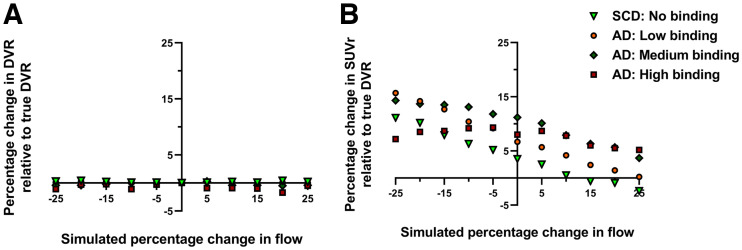
Percentage change in DVR (A) and SUVr (B) at 80–100 min relative to true DVR values as function of simulated flow changes for each binding condition.

Simulations revealed that under the SCD (almost no binding) and low-binding AD patient conditions, an inverse relation was observed; that is, with increasing flow, a decreasing bias for SUVr (with respect to true DVR) was observed (Fig. 4). A similar behavior was also observed under the medium-binding AD patient condition, but to a lesser extent. In the high-binding condition for AD patients, however, a relatively smaller effect of flow was observed on SUVr, implying that SUVrs remained relatively constant irrespective of the change in flow. In the case of DVR, no effect of flow was observed with any of the conditions (Fig. 4).

**FIGURE 5.**
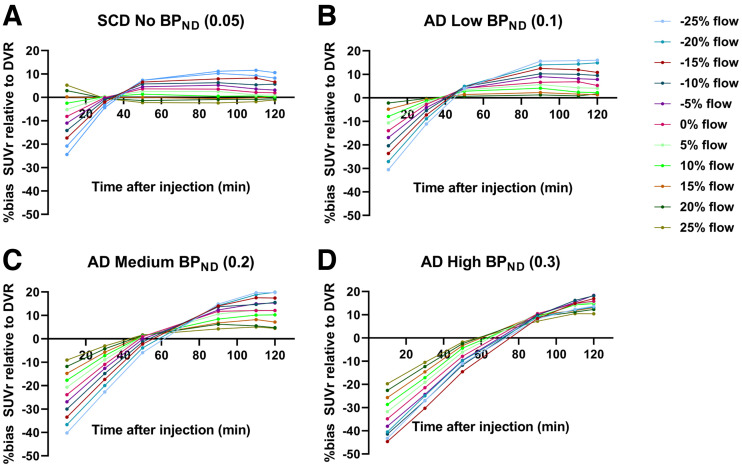
Percentage bias in SUVr relative to true DVRs as function of SUVr time intervals for simulated flow condition for SCD (almost no binding condition) (A), AD with low binding condition (B), AD with medium binding condition (C), and AD with high binding condition (D). Key illustrates different increases or decreases in flow.

On the basis of simulations, percentage bias in SUVr with respect to the true DVR varied with the choice of SUVr scanning interval and the underlying binding condition (Fig. 5). In general, SUVr overestimated DVR for all simulated R_1_ conditions from 80 min after injection; however, the impact of the change in flow on the directionality of the bias seems also to vary with respect to the choice of SUVr scanning interval (Fig. 5).

## DISCUSSION

We compared changes in ^18^F-flortaucipir specific binding using SUVr and DVR. In a 2-y longitudinal study, changes in ^18^F-flortaucipir DVR and SUVr were comparable in all patient groups. Only small changes in R_1_ occurred during this period, but these most likely contributed to the lack of difference between DVR and SUVr. However, simulations demonstrated marked differences between DVR and SUVr when large(r) changes in R_1_ were introduced. In addition, these differences between DVR and SUVr were shown to be dependent on the underlying level of tau pathology.

The most important finding in this study was the lack of major differences in the percentage change between ^18^F-flortaucipir DVR and SUVr in a 2-y observational study. Congruently, sample size calculations based on these data to inform future trials showed negligible differences between methods. Unlike a previous study using ^11^C-PiB (6), this finding indicates that ^18^F-flortaucipir SUVr provides an accurate estimate of change in specific binding in both patient groups. There are several possible reasons for the differences in findings between the 2 studies. First, an important factor contributing to our findings could be the relatively small or nonexistent R_1_ differences in this cohort. Previously, using ^11^C-PiB (6), we reported larger R_1_ changes in AD patients, which induced a large difference between SUVr and BP_ND_. This effect might perhaps indicate that ^11^C-PiB is more sensitive to changes in R_1_ than is ^18^F-flortaucipir. However, flow sensitivity may also depend on the scanning interval relative to tracer kinetics, as was seen previously for ^11^C-PIB (6). Similarly, this is the scenario for ^18^F-flortaucipir, and we therefore cannot directly compare the 2 tracers in this respect. Second, it has been reported that accumulation of tau pathology is a slowly developing process, with annual percentage changes of about 0.5%–3% in Aβ-positive cognitively unimpaired subjects and up to 3%–10% in Aβ-positive cognitively impaired subjects (34–37). The annual percentages change in the present study was generally comparable in SCD subjects (on average, 1.08% SUVr and 1.28% DVR) and slightly lower in AD subject (2.73% SUVr and 2.52% DVR). The test–retest repeatability of ^18^F-flortaucipir, as reported previously (38), lies at around 1.98% (0.78–3.58) for DVR and 3.05% (1.28–5.52) for SUVr at 80–100 min. Although the test–retest repeatability was significantly better for DVR (38), annual percentage changes as found in the present study still fall within 1 SD of the test–retest repeatability for both DVR and SUVr, suggesting that observed changes might be too small to detect differences between analytic methods. Finally, differences with respect to tracer target affinity, isotope (^11^C vs. ^18^F), and pharmacokinetic behavior might have introduced differences that caused the differences in results.

Currently, the effects of pharmacotherapeutic interventions on cerebral blood flow are unclear. Therefore, we performed simulations to investigate the impact of large(r) changes in relative cerebral blood flow/R_1_ on the accuracy of SUVr and DVR. The bias with SUVr relative to DVR was different for each flow condition, and this bias was additionally influenced by the underlying tau load, with decreasing bias in cases of low tau load/binding or constant bias for high tau load/binding. Depending on the underlying tau load, regional changes in flow resulted in variable changes in SUVr, which was not the scenario with DVR. Similar findings were previously observed using ^18^F-cyclofoxy (39).

On top of flow condition and the underlying tau load, the choice of SUVr time interval also effected the accuracy, which was again different for different binding conditions. A previous study found large positive biases for SUVr using different time intervals when compared with dynamic methods (8). Furthermore, Golla et al. (8) observed that the bias in SUVr for a specific scanning interval is not constant but is dependent on the underlying tau load and the choice of SUVr scanning interval. This has important implications, since scanning intervals for static protocols are often not strictly enforced; thus, deviations in scanning intervals between static and longitudinal scans are common. These discrepancies will increase variability and uncertainty, which will increase required sample sizes for SUVr. Differing underlying tau load in the sample studied will only increase the bias in SUVr further. It is worth noting that, in the current study, SUVr was extracted from the dynamically acquired data. In addition, scanning interval was strictly enforced in the context of the 2 scanning sessions within the dynamic protocol. For both these reasons, SUVr in this study was not affected by deviations in scanning, and the results may therefore be too optimistic in this respect.

The discrepancies between methods using simulations may have important implications for longitudinal ^18^F-flortaucipir studies and intervention studies. Our findings imply that SUVr is not the parameter of preference when large variations in blood flow are expected, although to what order of magnitude remains to be elucidated. A consideration to address when using repeated dynamic scans is potential selection bias, because severely affected patients might not be able to undergo such a demanding procedure. In patients with moderate to severe AD, this is indeed debatable. However, pharmacotherapeutic trials currently show a shift in target population, primarily including patients with mild, prodromal, or preclinical autosomal-dominant AD. Those patients can tolerate the longer dynamic scan procedures.

^18^F-flortaucipir is useful for investigating pathologic tau load differences between SCD subjects and AD patients. However, in an early-dementia cohort for which we do not expect specific binding in the neocortex, measurement of tau deposition shows large variability. Indeed, in such a sample, 64% of the cortical signal variability can be explained by off-target binding (40). Partial-volume correction does not completely explain the variability in the cortical signal. Therefore, the variability in the signal in cohorts with low tau deposition related to off-target binding should be considered when examining early tau deposition using ^18^F-flortaucipir.

## CONCLUSION

Static scanning protocols provide accurate estimates of specific ^18^F-flortaucipir binding in observational studies. Dynamic scanning protocols and fully quantitative data analysis methods are preferred when large(r) flow changes in the brain are expected (such as in later disease stages or pharmacotherapeutic interventions). Use of semiquantitative methods in such conditions carries the inherent risk that potential effective therapeutic interventions are discarded, especially when expected effect sizes are small.

## DISCLOSURE

Research at the Amsterdam Alzheimer Center is part of the neurodegeneration program of Amsterdam Neuroscience; the Amsterdam Alzheimer Center is supported by Alzheimer Nederland and Stichting VUmc funds. ^18^F-flortaucipir PET scans were made possible by Avid Radiopharmaceuticals Inc. This study was funded by a ZonMW Memorabel grant. Wiesje Van der Flier holds the Pasman chair and received grant support from ZonMW, NWO, EU-FP7, Alzheimer Nederland, CardioVascular Onderzoek Nederland, Stichting Dioraphte, Gieskes-Strijbis Fonds, Boehringer Ingelheim, Piramal Neuroimaging, Roche BV, Janssen Stellar, and Combinostics. All funding is paid to the institution. Bart van Berckel has received research support from EU-FP7, CTMM, ZonMw, NOW, and Alzheimer Nederland. Bart van Berckel has performed contract research for Rodin, IONIS, AVID, Eli Lilly, UCB, DIAN-TUI, and Janssen; was a speaker at a symposium organized by Springer Healthcare; has a consultancy agreement with IXICO for the reading of PET scans; is a trainer for GE; and receives financial compensation only from Amsterdam UMC. No other potential conflict of interest relevant to this article was reported.

## Notes

KEY POINTS **QUESTION:** How do the semiquantitative (SUVr) and quantitative (R_1_, BP_ND_) parameters of longitudinal ^18^F-flortaucipir PET scans, and their vulnerability to changes in blood flow, compare in subjects along the AD continuum? **PERTINENT FINDINGS:** In a 2-y longitudinal ^18^F-flortaucipir PET study including 38 **s**ubjects with SCD and 24 patients with AD, relative cerebral blood flow changes (R_1_) were small, and semiquantitative (SUVr) and quantitative (BP_ND_) parameters yielded highly similar estimates of specific binding. However, simulations showed that large(r) flow changes may potentially affect ^18^F-flortaucipir SUVr. **IMPLICATIONS FOR PATIENT CARE:** Given that it is currently unknown to what order of magnitude pharmacotherapeutic interventions may induce changes in cerebral blood flow, caution may be warranted when changes in flow are large(r), and DVR/BP_ND_ may be preferred under such conditions to ensure representative quantification of ^18^F-flortaucipir PET images.
